# Backpressure Optimization in Foam Injection Molding: Method and Assessment of Sustainability

**DOI:** 10.3390/polym12112696

**Published:** 2020-11-16

**Authors:** Clemens Kastner, Thomas Mitterlehner, Dominik Altmann, Georg Steinbichler

**Affiliations:** 1 Competence Center CHASE GmbH, Altenberger Strasse 69, A-4040 Linz, Austria; 2 Institute of Polymer Injection Molding and Process Automation, Johannes Kepler University Linz, Altenberger Strasse 69, A-4040 Linz, Austria; thomas.mitterlehner@jku.at (T.M.); dominik.altmann@jku.at (D.A.); georg.steinbichler@jku.at (G.S.); 3 Kompetenzzentrum Holz GmbH (Wood K plus)—Biobased Composites and Processes, Altenberger Strasse 69, A-4040 Linz, Austria

**Keywords:** polymer processing, foam injection molding, polypropylene, optimization, sustainability, backpressure

## Abstract

Inspired by the Industry 4.0 trend towards greater user-friendliness and self-optimization of machines, we present a novel approach to reducing backpressure in foam injection molding. Our method builds on the compressibility of polymer-gas mixtures to detect undissolved gas phases during processing at insufficient backpressures. Identification of a characteristic behavior of the bulk modulus upon transition from homogeneous to heterogeneous polymer-gas mixtures facilitated the determination of the minimum pressure required during production to be determined, as verified by ultrasound measurements. Optimization of the pressure conditions inside the barrel by means of our approach saves resources, making the process more sustainable. Our method yielded a 45% increase in plasticizing capacity, reduced the torque needed by 24%, and required 46% less plasticizing work and lower pressures in the gas supply chain. The components produced exhibited both improved mechanical bending properties and lower densities. From an economic point of view, the main advantages of optimized backpressures are reduced wear and lower energy consumption. The methodology presented in this study has considerable potential in terms of sustainable production and offers the prospect of fully autonomous process optimization.

## 1. Introduction

Against the backdrop of greenhouse gas emissions and the concomitant climate change, sustainability has become a central objective [1,2]. In the past decades, polyolefins—and of the semi-crystalline types, especially polypropylene (PP) and polyethylene (PE) [3]—have become increasingly important in material sciences. From a sustainability point of view, their use in a myriad of applications can be explained by (i) their economic, energy-saving production and processing, which is mainly due to the low melt or softening temperatures required; (ii) their significant role in lightweight functional design [4] (i.e., the integration of multiple functions into a single component); and (iii) their low density and, thus, light weight. Especially in transportation, plastics help to reduce weight and thus also fuel consumption and emissions. Broadly, a 10% decrease in vehicle weight can reduce fuel consumption by 5% [5].

The most important processing technology for polymeric materials is injection molding [6,7]. Continuous improvement to keep up with market requirements has led to the development of numerous specialized injection molding technologies, some of which facilitate more sustainable production, while others allow production of components with new structures and properties. Uniquely, foam injection molding holds the potential to combine both.

This technology incorporates gases (mostly nitrogen or carbon dioxide) that are finely dispersed and dissolved in the polymer melt during production [8,9]. Foam injection molding technologies are subdivided into physical and chemical processes. The former work with direct injection of pure gases in supercritical state [10], while the latter use chemical masterbatches that decompose upon heating during plasticization. This work concentrates on physical foaming. Upon injection into the cavity of the mold, the polymer-gas mixture experiences a pressure drop, which leads to thermodynamic instability [11] and causes the gas to change back to the gaseous state. The result is a component that exhibits compact outer layers and a foamed core, resembling a sandwich structure known from composite lightweight design. On the basis of pioneering work at the Massachusetts Institute of Technology (MIT) on the application of supercritical fluids (SCF), cell densities in the range of 10^9^ cells/cm^3^ have become possible [12].

Their mechanical characteristics (i.e., high bending stiffness in combination with low density and therefore great potential for light weight) are just one of the advantages of foam-injection-molded components. As they consist of only one constituent, they are easily recyclable. Gases dissolved in polymer melts reduce melt viscosity during processing. Reductions by more than 50% have been achieved using carbon dioxide (CO_2_) [13,14]. This technology also facilitates product properties, such as acoustic and/or thermal insulation or improved warpage behavior [15,16,17,18]. Foam injection molding enables energy and resource-efficient processing and saves energy throughout the product life cycle. These benefits have led to numerous industrial applications in the automotive industry, insulation appliances, the sporting goods industry, construction, and electronic components [19,20,21].

Every foaming process involves the uptake of gases by the polymer melt. For physical incorporation of gases into the polymer melt during processing, high pressures (in combination with intense shearing) are favorable [22,23]. Insufficient pressures lead to undissolved (and, therefore, discrete), nonuniformly distributed gas phases, which negatively affects surface appearance [24] and mechanical properties of the components produced, and can cause process instabilities. However, from a processing point of view, excessive pressures slow conveying, increase energy consumption, and promote abrasive wear on screw and cylinder. As can be seen, the backpressure during processing of gas-containing polymer melts is crucial to the sustainability of the process.

In (foam) injection molding, the backpressure (i.e., the pressure exerted on the polymer melt in front of the screw) during plasticization is controlled and kept above a predefined value. The time for the uptake of gases is usually limited to seconds or minutes. Therefore, backpressures in foam injection molding are approximately one order of magnitude greater (amounting to up to 200 bar) than in standard injection molding.

Although the backpressure plays a crucial role, only few studies have been published on its influence on process or product. Kharbas et al. [22] investigated the effects of process conditions on the resulting mechanical properties of a foam-injection-molded polyamide 6 nanocomposite. An increase in backpressure led to optimized tensile properties. They argued that higher backpressures caused greater pressure-drop rates and, thus, finer cell structures. Guo et al. [25] studied the effect of experimental conditions on the cell structure of PP nanocomposite foams. An increase in backpressure from 0 to 15 MPa caused a decrease in cell density, a corresponding increase in cell size and enhanced dissolution. Volpe and Pantani [23] investigated the foaming of polylactide (PLA) with N_2_. They found a decrease in injected material mass (at constant volume) and density reductions by up to 25% for reduced backpressures. Further, the absolute flexural modulus as well as the normalized elastic modulus (i.e., the ratio between modulus and density) increased with increasing backpressure. A study by Chien et al. [26] confirmed these results for PP. Tensile and flexural strength, stiffness and component weight increased with higher backpressures. In their analysis, Rizvi and Bhatnagar [27] also focused on the microstructure of polystyrene foamed with N_2_. In general, high backpressures cause an increase in the amount of gas that can be dissolved in the melt, which, in turn, results in smaller cells. They identified an optimum range for this process parameter and highlighted its great importance.

To the best of our knowledge, to date no study has focused on backpressure optimization. We therefore present a novel method for optimizing the backpressure in a foam injection molding process. Our approach is based on a recently developed measurement method [28] that uses the compressibility of the polymer-gas mixture to enable in-line detection of dynamic solubility limits in foam injection molding (a brief outline of set-up and test procedure for this underlying measurement method is provided in the upcoming chapter). For given process conditions, it allows determination of the lowest backpressure necessary for generating a homogeneous polymer-gas solution. This optimization procedure can be conducted within the machine without any additional equipment. It was tested in a conventional foam injection molding process using both nitrogen and carbon dioxide in a manual procedure. For verification of the method, ultrasound measurements were taken.

In addition to very good machine performance, beneficial effects on the mechanical properties of the components produced were observed. Most importantly, our approach reduces energy consumption considerably and, therefore, improves sustainability of the whole process. Our method represents a significant step towards a more ecologically-sustainable foaming process. In line with Industry 4.0, ongoing further developments should result in a self-optimizing system.

## 2. Theoretical Background

Our fundamental idea was to use the compressibility of polymer–gas mixtures to describe the amount of gas incorporated into the polymer melt. Numerous theories are available that describe mathematically the compression behavior of such mixtures. For the initial theoretical description of the *pvT* behavior, the Sanchez–Lacombe equation of state [29,30] (SL-EOS) was applied. Each pure fluid can be fully described by reduced density, pressure and temperature, ${\tilde{\rho }}_{i}$, ${\tilde{p}}_{i}$, and ${\tilde{T}}_{i}$, which can in turn be calculated from the corresponding characteristic parameters, $\rho_{i}^{*}$, $p_{i}^{*}$, and $T_{i}^{*}$, according to:

(1) $$\tilde{T}=\frac{T}{T^{*}},\;\tilde{p}=\frac{p}{p^{*}},\;\tilde{\rho }=\frac{\rho }{\rho^{*}}.$$

These characteristic parameters are obtained from experimental *pvT* data of the fluid to be described.

The SL-EOS allows this description of pure fluids to be extended. Using the characteristic parameters of two different fluids, mixing rules can be applied (for a detailed description of mixing rules see [30]). This enables derivation of the characteristic parameters $\rho_{m}^{*}$, $p_{m}^{*}$, and $T_{m}^{*}$, and of the corresponding reduced versions, ${\tilde{\rho }}_{m}$, ${\tilde{p}}_{m}$, and ${\tilde{T}}_{m}$, for a mixture of these two fluids. The SL-EOS is then given by:

(2) $${\tilde{\rho }}_{m}^{2}+{\tilde{p}}_{m}+{\tilde{T}}_{m}\left[\ln\left(1-{\tilde{\rho }}_{m}\right)+\left(1-\frac{1}{r_{m}}\right)\rho_{m}\right]=0.$$

The molecular parameter, $r_{m}$, can be calculated from the characteristic parameters, the molecular mass, $M_{i}$, and the molar fraction, $x_{i}$, of each fluid in the mixture and the universal gas constant, R, according to:

(3) $$r_{m}=\frac{1}{R}\underset{i}{\sum }\frac{M_{i}p_{i}^{*}x_{i}}{T_{i}^{*}\rho_{i}^{*}}.$$

The method developed in this work uses the bulk modulus, C, (i.e., the reciprocal compressibility, 1/β) of polymer–gas mixtures, which relates a pressure change, ∆p, to a relative change in volume, $\Delta V/V_{0}$:

(4) $$C=-\frac{\Delta p}{\Delta V}V_{0}.$$

**Figure 1:**
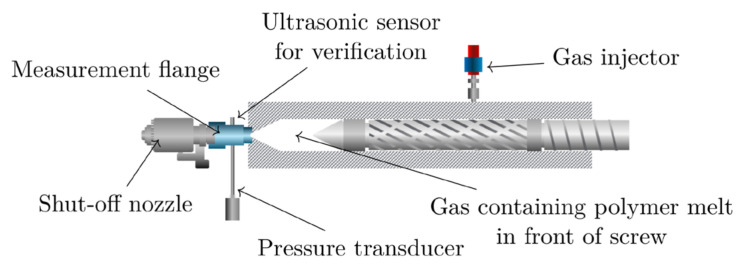
Schematic representation of the plasticizing unit on an injection molding machine equipped for solubility measurements (shut-off nozzle and gas injector are standard equipment for foam injection molding; measurement flange, pressure transducer, and ultrasonic sensor are mounted specifically for the purpose of bulk modulus measurements).

To calculate these parameters with values obtained from an injection molding machine, the following equation can be used:

(5) $$C=-\frac{\Delta p}{\Delta S}\left(\frac{V_{M}}{A}+S_{0}\right),$$

where S is the axial screw position, A the cross section of the cylinder, and $V_{M}$ the volume in front of the non-return valve when the screw is in its foremost position. Using standard pressure values from the machine, the bulk modulus is given in bar. A schematic representation of the set-up for such bulk modulus measurements is given in Figure 1. For the realization of a pressure change in combination with a change in volume, the shut-off nozzle initially remains closed as the injection stage starts (i.e., the screw starts to move forward). Once a predefined maximum injection pressure is reached, the screw comes to rest. The change in pressure and in axial screw position during this compression stage are measured and used for the calculation of the bulk modulus according to Equation (5). After that, the shut-off nozzle opens, and the melt is injected into the cavity.

**Figure 2:**
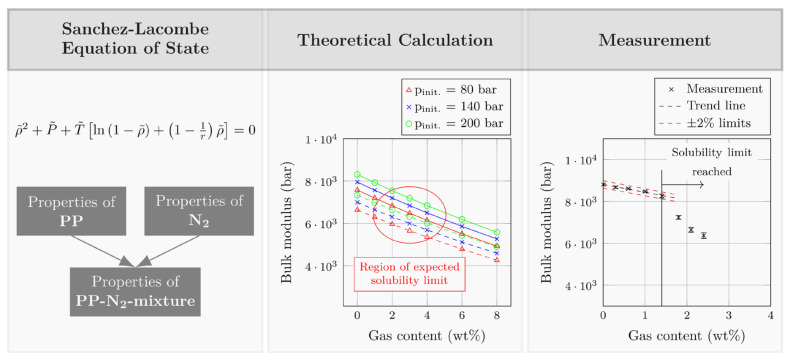
Workflow for identifying the compression behavior of polymer–gas mixtures with excessive gas content.

Initial considerations focused on the bulk modulus behavior for excessive amounts of gas. According to the literature, the bulk modulus of a polymer melt decreases with increasing amount of dissolved gas [31]. The assumption was that, at gas contents beyond the solubility limit, an undissolved gas phase would be present in the polymer melt. Since the bulk modulus of the separate gas phase is lower than that of the surrounding polymer, the bulk modulus of the polymer–gas mixture should drop sharply once a threshold gas content has been exceeded. Figure 2 summarizes the workflow for identifying, describing, and characterizing the material response to large and, ultimately, excessive gas content. Based on the SL-EOS, the bulk modulus was described for a mixture of PP and nitrogen (N_2_). As can be seen, the calculation can describe a continuous decrease in bulk modulus, but not the transition from homogeneous solution to heterogeneous mixture. Measurements using the same material combination (i.e., PP and N_2_) at a constant backpressure of 140 bar, however, eventually showed this transition.

The bulk modulus of polymer–gas mixtures can, therefore, be used to accurately identify a second, discrete gas phase in the screw antechamber and thus the dynamic solubility limit. Figure 2 shows that this limit was reached by gradually increasing the gas content at constant process parameters (temperature, pressure, screw speed, etc.). Conversely, the gas content can also become too high when the pressure drops below a critical value (cf. Henry’s law of solubility of gases in polymers [32]). In this case, a plot of the bulk modulus against backpressure at constant gas content should exhibit similar behavior, which means that the modulus should drop once the pressure has fallen below a lower limit.

## 3. Materials and Methods

Although backpressure during plasticization is an important parameter in foam injection molding, it is commonly set based on experience. As no empirical process characteristics for selecting a defined backpressure have been found to date, a reduction is usually possible while maintaining a homogeneous gas-polymer solution during production. Since the bulk-modulus approach can detect an undissolved gas phase, it can be used to find a minimum necessary backpressure (MNB) that still ensures homogeneity during production.

### 3.1. Optimization Procedure

The optimization algorithm developed in this work gradually reduces the backpressure while keeping all other parameters (including gas content) constant. After each reduction, at least 15 consecutive shots were performed to reach an equilibrium state before the bulk modulus was measured in five further shots. Only one constraint was predefined: Plasticizing time (and thus cycle time) was to be kept constant, which required adaptation of the screw speed.

According to Henry’s law, and as described above, lower pressures reduce the amount of gas that can be dissolved in the polymer matrix. Translated to the injection molding process, a reduction in backpressure reduces the amount of gas that can be added to the melt. As with standard dynamic solubility measurements (Figure 2), a drop in the bulk modulus curve at a particular backpressure indicates the transition from homogeneous to heterogeneous polymer-gas mixture. A pressure slightly above this transition point is then regarded as sufficient.

### 3.2. Materials and Equipment

In this work, the optimization was applied to two polymer-gas combinations (PGC), as listed in Table 1. Both polymer grades were used as received from Borealis Polyolefine GmbH (Linz, Austria). A standard additive package was used for both materials. The unfilled PP homo(PP-H)- and co(PP-C) polymers have melt flow rates of 75 g/10 min and 18 g/10 min (230 °C, 2.16 kg), respectively, which allows for an assessment of the applicability of the optimization method to different materials. 15% talc was added as a nucleation agent. Experiments with PGC 1 were conducted at an Engel DUO 700 injection molding machine from Engel Austria GmbH (Schwertberg, Austria). For PGC 2, an Engel DUO 1500 injection molding machine was used. The reason for the usage of a different machine for PGC 2 is that the first one (DUO 700) is incompatible with the SCF delivery unit for CO_2_. Both machines were equipped with a MuCell-screw geometry and a SCF delivery unit from Trexel, Inc. (Wilmington, MA, USA). A shut-off nozzle was provided by Herzog Systems AG (Flawil, Switzerland). For mechanical characterizations, plates (800 × 400 mm) with a thickness of 2.7 mm were produced. Specimens for flexural tests were milled in accordance with DIN EN ISO 178 in the flow direction.

## 4. Results and Discussion

### 4.1. Backpressure Optimization Using N_2_

**Figure 3:**
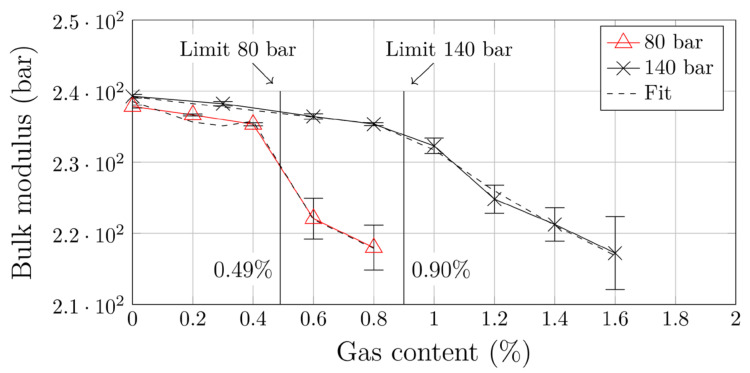
Reference dynamic solubility limits obtained for PGC 1.

It is crucial to understand that unwanted discrete gas phases can appear because of insufficient pressures or excessive gas contents and that minimum pressure and maximum gas content are interdependent. Therefore, prior to pressure optimization using PGC 1, dynamic solubility limits at backpressures of 80 and 140 bar were determined as reference values. The corresponding bulk modulus curves are shown in Figure 3. At 80 bar and 140 bar, the solubility limits measured were 0.49% and 0.9%, respectively (a detailed explanation of the mathematical procedure for defining solubility limits can be found in [28]). Subsequently, two gas contents were defined for which the backpressure in the foaming process was to be optimized: 0.6% and 1.2%. Based on the results presented in Figure 3, a backpressure between 80 and 140 bar is necessary for 0.6% N_2_, and more than 140 bar for 1.2% N_2_.

**Figure 4:**
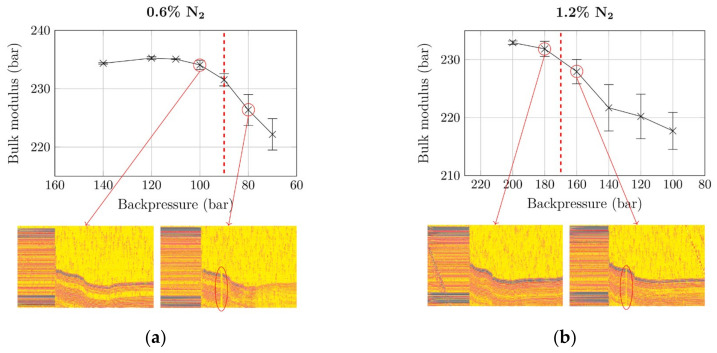
Development of bulk moduli with decreasing backpressure and ultrasound measurements for verification with (**a**) 0.6% N_2_ and (**b**) 1.2% N_2_. The interruptions on the ultrasound signals stem from an undissolved gas phase (i.e., heterogeneous polymer-gas mixture). Dashed vertical line: MNB.

The optimization procedure started at backpressures that are sufficiently high for homogenization of the material. Bulk modulus curves for subsequent gradual reduction in backpressure (according to the optimization procedure described above) were recorded and are presented in Figure 4. For calculating the bulk moduli, the specific injection pressure of the injection molding machine was used. The x-axes are reversed, as experiments were also conducted from high to low pressures.

The bulk moduli obtained at the initially set backpressure (140 bar for 0.6% N_2_; 200 bar for 1.2% N_2_) were among the highest in the whole optimization. This implies that these pressures were well above the minimum required. In the case of 0.6% N_2_, the bulk modulus formed a plateau at approximately 234.5 bar for backpressures down to 100 bar, which suggests unchanged homogeneity of the polymer–gas mixture. Below this pressure, the bulk modulus started to drop sharply. Similar behavior was observed for the higher gas concentration of 1.2% N_2_. At a backpressure of 200 bar, the largest bulk modulus with 232 bar was measured. As can be seen, the drop in the bulk modulus curve occurred at backpressures between 180 and 160 bar. In addition to the overall trends of the bulk modulus curves, the standard deviation is particularly interesting: It increases once the modulus curve starts to drop (i.e., when the backpressure becomes insufficient). For 0.6% N_2_, the standard deviation increased tenfold from 0.13% to 1.2% when the backpressure fell below the MNB. For 1.2% N_2_, the standard deviation increased from 0.35% to 1.48%.

To verify the bulk modulus procedure, we simultaneously performed ultrasound measurements in the measurement flange (see. Figure 1) during injection of the melt into the cavity. Plots of measurements just above and below the MNB are included in Figure 4 (each ultrasound measurement represents a time of approx. 2 s): Ultrasound signals recorded during injection molding cycles at sufficient backpressure (100 bar for 0.6% N_2_; 180 bar for 1.2% N_2_) are continuous, which implies a perfectly homogeneous melt. Interruptions, as observed at insufficient backpressures (marked by red ellipses), are due to discrete gas phases (i.e., small gas bubbles) that pass the ultrasound sensor, and are a clear sign of heterogeneity. As explained above: Insufficient backpressure during plasticization prevent the total amount of gas from being dissolved; a small portion of it remains undissolved. We thus confirmed the ability of our bulk-modulus-based method to accurately quantify the MNB.

### 4.2. Backpressure Reduction Using CO_2_: Mechanical Characterization

Based on the method developed, the influence of pressure optimization on mechanical properties of the produced components was investigated. Flexural tests are standard in the mechanical characterization of structural foams, which often have superior specific flexural properties (e.g., specific stiffness [33]). In our tests, we evaluated Young’s modulus and flexural strength.

**Figure 5:**
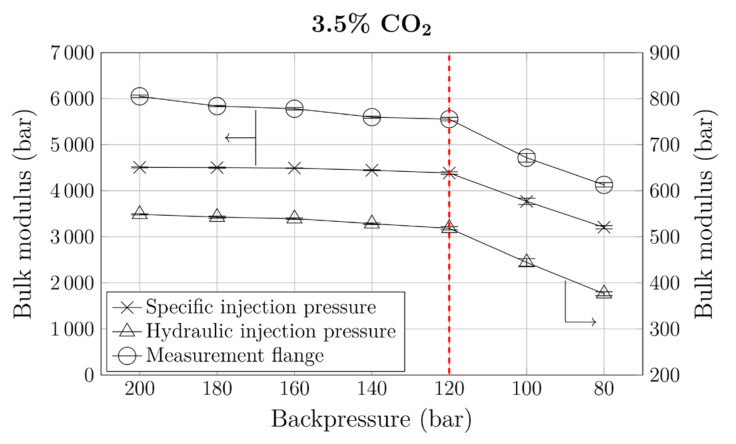
Development of bulk moduli with decreasing backpressure for 3.5% CO_2_. Dashed vertical line: MNB.

First, the pressure was optimized. As mentioned above, one constraint was to perform these optimizations at constant cycle times, which required reduction of the screw speed after each pressure reduction. For optimization, a CO_2_ content of 3.5% was chosen. This amount was again based on prior measurements of dynamic solubility, which indicated that backpressures between 80 and 140 bar were required. To ensure that the optimization started at a sufficient backpressure, a high initial pressure of 200 bar was chosen. The result of the backpressure optimization using the bulk-modulus method are presented in Figure 5. To better understand the capabilities of the bulk-modulus method, we considered three different pressures (specific and hydraulic injection pressure and pressure in the measurement flange) in a series of tests. As the results show, using a different pressure value only shifts the curves to different absolute values, while their shape remains unchanged. This makes both the pressure transducer in the measurement flange and the measurement flange itself (as shown in Figure 1) obsolete and simplifies the method. All three pressures yielded the same results: The bulk moduli of the polymer-gas mixtures formed a plateau for backpressures down to 120 bar. Pressures below this threshold value led to a sudden decrease in bulk modulus. This change in slope indicates a heterogeneous polymer-gas mixture and, thus, insufficient backpressure. The backpressure optimization yielded 120 bar as an optimum.

**Figure 6:**
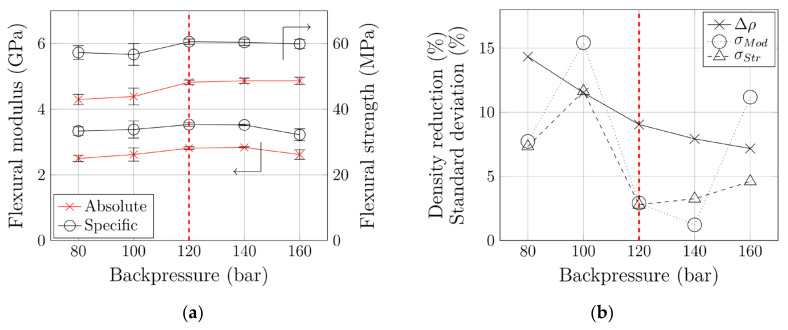
(**a**) Flexural properties of foamed PGC 2 and (**b**) assessment of potential for light weight and process stability at various backpressures. Dashed vertical line: MNB.

The subsequent mechanical characterization not only focused on flexural properties, but also included density reduction, Δρ, and standard deviations of modulus, and strength measurements, $\sigma_{Mod}$ and $\sigma_{Str}$, respectively. Each measurement was carried out using five specimens milled from plates from 5 consecutive shots. The results are summarized in Figure 6. In agreement with the literature [22,23,26], our work showed that larger backpressures have a positive influence on mechanical properties. However, we additionally found that excessive backpressures have a negative effect on the mechanical performance and that optimized backpressure slightly above the MNB is favorable for both mechanical properties (i.e., Young’s modulus and flexural strength). This emphasizes that the process window is narrow and that the backpressure is an important process parameter.

Our backpressure optimization (i.e., the reduction from 160 to 120 bar) caused an increase by 7.4% (absolute) or 9.6% (specific) in flexural modulus. Choosing an insufficient backpressure of 80 bar resulted in a decrease by 11.1% (absolute) or 5.6% (specific) compared to the values at the optimum backpressure of 120 bar. Flexural strength was unaffected by backpressure optimization. A reduction to 80 bar and the heterogeneity thereby induced led to a reduction in mechanical performance by 10.9% (absolute) or 5.4% (specific).

In transportation in particular, the potential for light weight (i.e., density reduction) is of interest. It causes the differences in changes in absolute and specific flexural properties described above. Note that the dosing stroke of the screw (i.e., the volume of plasticized polymer melt) was kept constant. Consequently, larger pressures exerted on the melt led to higher compression, which in turn influenced the density of the parts produced. A density reduction from 7% to 9% was achieved. The combination of lower density and improved mechanical performance as a result merely of reducing backpressure and screw speed (all other process parameters were kept constant) underlines the significant potential of this optimization.

Very interesting behavior was observed in terms of standard deviation during mechanical characterization. As Figure 6 shows, both $\sigma_{Mod}$ and $\sigma_{Str}$ reached a minimum at optimized backpressures. The standard deviation of flexural modulus measurements decreased from 11.2% to 2.9% due to backpressure optimization. With a standard deviation of 15.4%, the poorest reproducibility was observed at an (insufficient) backpressure of 100 bar. Flexural strength measurements showed similar behavior: The standard deviation decreased from 4.6% to 2.8% when the backpressure was reduced to 120 bar, and also reached its maximum (11.6%) at an insufficient backpressure of 100 bar. This means that the reproducibility of the foam injection molding process improves dramatically when the backpressure is optimized. Both excessive pressures during material provision and insufficient pressures (i.e., heterogeneous polymer-gas mixture) degrade process stability and make part quality inconsistent.

In summary, optimization of the backpressure also optimizes numerous other parameters: Flexural modulus and flexural stiffness were increased, and density reduced. Standard deviation during mechanical characterization was minimized, which means that process stability was improved significantly.

### 4.3. Backpressure Reduction Using CO_2_: Assessment of Sustainability

**Figure 7:**
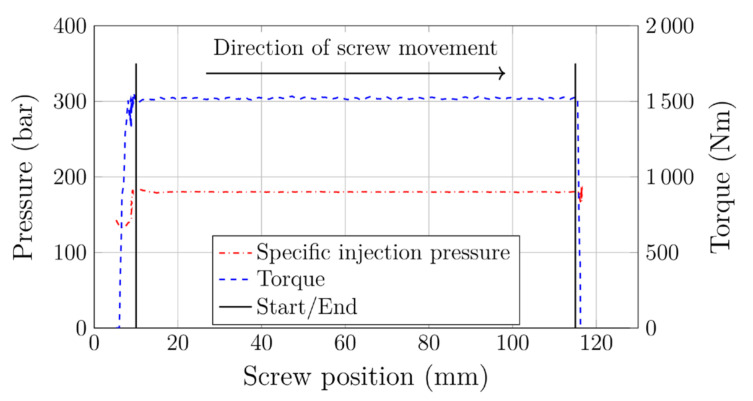
Specific injection pressure and torque within a dosing stage.

In addition to improving mechanical and process performance, the reduction in backpressure during plasticization enables more sustainable processing. During optimization we monitored various process characteristics across the whole range of pressures, focusing on the dosing stage. Throughout the dosing stage, the backpressure (and thus also the specific injection pressure) was kept constant by the machine control. This also kept the torque virtually constant. As an example, Figure 7 includes specific injection pressure and torque throughout the dosing stage of one (arbitrarily chosen) injection molding cycle. Based on these parameters, we quantified the influence of backpressure on overall process sustainability.

As explained earlier, one constraint during optimization was to keep cycle time constant. Consequently, the screw speed, *n*, had to be adapted after each pressure reduction to keep the dosing time, $t_{d}$, constant. In addition to the torque, M, in the first step the area-specific plasticizing work, $W_{pl,spec}$, was calculated by integrating the pressure curve according to:

(6) $$W_{pl,spec}=\int_{s_{1}}^{s_{2}}p_{spec}\left(s\right)ds,$$

where $p_{spec}$ is the specific injection pressure and $s_{1}$ and $s_{2}$ represent the screw positions at the beginning and the end of the dosing stroke, respectively (marked by vertical dashed lines in Figure 7).

Another approach, suggested by Mount III [34], allows the plasticizing power, $P_{pl}$, to be calculated:

(7) $$P_{pl}=2\pi Mn.$$

The corresponding plasticizing work, $W_{pl}$, is obtained by:

(8) $$W_{pl}=P_{pl}t_{d}.$$

**Figure 8:**
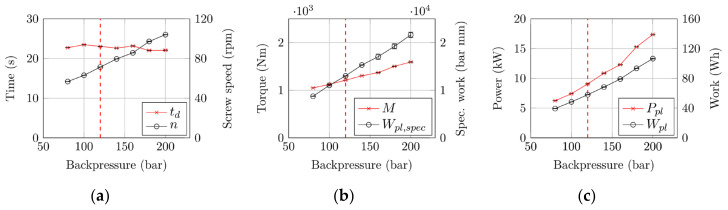
Relationships between various performance parameters and backpressure during plasticization for assessment of sustainability potential: (**a**) Plasticizing time, screw speed; (**b**) torque, area-specific injection work; and (**c**) plasticizing power, plasticizing work.

The results are summarized in Table 2 and illustrated in Figure 8.

Linear correlations were found between the backpressure applied and the process parameters monitored. The reference values for the following discussion of the improvements we achieved are the maximum (initial) backpressure of 200 bar and the optimized one at 120 bar (see Figure 5).

The backpressure acts in the screw antechamber. As its effect is directed against the flow direction of the melt, lower backpressures improve the conveying behavior of the plasticizing unit, as shown in Figure 8a. In order to produce at a constant dosing time (average: 22.7 s, standard deviation: 0.54 s), the screw speed had to be reduced by 31%, which corresponds to an increase in plasticizing capacity by 45%. As a consequence, the resulting torque and area-specific injection work (see Figure 8b) were reduced by 24% and 40%, respectively. The reductions in plasticizing power and plasticizing work—both by 46%, because they are related to each other via the constant dosing time—were even more substantial. These results clearly show the noteworthy sustainability potential that the backpressure optimization we have presented offers. However, it is not only the substantial reduction in energy consumption that makes the optimization valuable for producers. A reduction in backpressure during plasticization also means less wear on screw and cylinder, material-friendly processing (e.g., less fiber breakage) or reduced cavity pressures.

## 5. Conclusions

We have presented a novel optimization procedure that is based on the compressibility of polymer–gas mixtures and allows the backpressure for successful plasticization to be minimized.

We developed the method using a commercial PP homopolymer in combination with nitrogen as a blowing agent. First, dynamic solubility limits of 0.49% and 0.9% N_2_ at pressures of 80 and 140 bar were determined. Based on these preliminary tests, gas contents of 0.6% and 1.2% were chosen. Application of the backpressure optimization using our bulk-modulus method yielded the minimum necessary backpressures of 90 and 170 bar, respectively (verified by ultrasonic measurements).

Similar investigations using a commercial PP copolymer in combination with carbon dioxide confirmed the capability of our optimization procedure and yielded an optimized backpressure of 120 bar at a gas concentration of 3.5%. Additionally, flexural modulus and strength, as well as density reduction and process stability, were investigated. Backpressure optimization maximized mechanical properties and minimized density and process instabilities. The greatest potential of this pressure optimization, however, was found to be the increase in production sustainability: it resulted in 46% less plasticizing work and power, a 31% reduction in screw speed (i.e., a 45% increase in conveying capacity), and 24% less torque. In addition to this immense reduction in energy consumption, the foam injection molding process benefits from less wear, material-friendly processing (e.g., less fiber breakage) or reduced cavity pressures.

Future work will concentrate on the automation of this optimization procedure and the creation of a graphical user interface. The ultimate objective is an autonomous, self-optimizing injection molding machine that meets current visions of Industry 4.0. The presented method has the potential for a user-friendly foam injection molding process in which the users need only specify the desired gas content; the machine relieves the user from all other responsibilities.

## 6. Patents

The bulk modulus method used in this work is patented and published as AT520733A4, AT520733B1, CN110394956A, DE102019108997A1, KR20190124652A, US2019329470A1.

## Figures and Tables

**Table 1:** Materials used in this work and their application.

.	PP-H	PP-C	Blowing Agent	Application
**PGC 1**	-	85%	N_2_	Method development, verification
**PGC 2**	85%	-	CO_2_	Mechanical characterization, sustainability potential

**Table 2:** Various performance parameters for assessing solubility during backpressure optimization.

Backpressure	(bar)	80	100	120	140	160	180	200
$t_{d}$	(s)	22.73	23.46	23.05	22.62	23.15	22.03	22.08
n	(rpm)	56.76	63.06	71.37	79.58	85.74	97.15	104.06
M	(Nm)	1049	1123	1212	1303	1370	1502	1593
$W_{pl,spec}$	(bar·mm)	8738	11,036	13,020	15,301	17,051	19,223	21,627
$W_{pl}$	(Wh)	39.37	48.34	58.00	68.25	79.08	93.50	106.48
$P_{pl}$	(W)	6.24	7.42	9.06	10.86	12.30	15.28	17.36
